# Prioritising between direct observation of therapy and case-finding interventions for tuberculosis: use of population impact measures

**DOI:** 10.1186/1741-7015-4-35

**Published:** 2006-12-20

**Authors:** Richard F Heller, Islay Gemmell, Richard Edwards, Iain Buchan, Shally Awasthi, James A Volmink

**Affiliations:** 1 Evidence for Population Health Unit, University of Manchester, Oxford Road, Manchester M13 9PT, UK; 2 University of Otago, New Zealand; 3 King George's Medical College, Lucknow, India; 4 Faculty of Health Sciences, University of Cape Town, South Africa

## Abstract

**Background:**

Population impact measures (PIMs) have been developed as tools to help policy-makers with locally relevant decisions over health risks and benefits. This involves estimating and prioritising potential benefits of interventions in specific populations. Using tuberculosis (TB) in India as an example, we examined the population impact of two interventions: direct observation of therapy and increasing case-finding.

**Methods:**

PIMs were calculated using published literature and national data for India, and applied to a notional population of 100 000 people. Data included the incidence or prevalence of smear-positive TB and the relative risk reduction from increasing case finding and the use of direct observation of therapy (applied to the baseline risks over the next year), and the incremental proportion of the population eligible for the proposed interventions.

**Results:**

In a population of 100 000 people in India, the directly observed component of the Directly Observed Treatment, Short-course (DOTS) programme may prevent 0.188 deaths from TB in the next year compared with 1.79 deaths by increasing TB case finding. The costs of direct observation are (in international dollars) I$5960 and of case finding are I$4839 or I$31702 and I$2703 per life saved respectively.

**Conclusion:**

Increasing case-finding for TB will save nearly 10 times more lives than will the use of the directly observed component of DOTS in India, at a smaller cost per life saved. The demonstration of the population impact, using simple and explicit numbers, may be of value to policy-makers as they prioritise interventions for their populations.

## Background

Tuberculosis (TB) is a major global public health problem. Reducing the burden of morbidity and mortality due to TB relies on identifying the treatments and preventive measures of greatest impact, and communicating this information to decision-makers so that the most appropriate policies are implemented.

We have previously described a series of population impact measures (PIMs) to describe the population impacts of risks and benefits [1-3]. These are measures of absolute risk and are based on previous methods for describing clinical and population impacts. They provide local meaning to an otherwise confusing mixture of generic evidence and local statistics, helping policy-makers to identify and prioritise the potential benefits of interventions in their own population. PIMs are simple to compute, and contain the elements to which policy-makers need to pay attention in the commissioning or improvement of services. They are designed as useful additions to the generic measures of illness burden, from which they differ in having event-specific outcomes rather than generic outcomes such as the quality-adjusted life year (QALY) or the disability-adjusted life year (DALY).

The World Health Organization (WHO) has promoted the Directly Observed Treatment, Short-course (DOTS) strategy, which comprises five elements, (political commitment, case detection using sputum microscopy, standardised short-course chemotherapy, regular drug supply, and a standardised recording and reporting system). These elements incorporate direct observation of treatment but exclude active case finding [4]. DOTS programmes may differ in the emphasis placed on the five elements, and may incorporate additional features[5]. In India, the Revised National Tuberculosis Control Programme gives considerable emphasis to directly observed treatment, so that it is now one of the five main elements [6]. Some authors have suggested that case finding is underemphasised as a global TB control strategy [7-11], and that directly observed therapy may be overemphasised[5,12]. Case-finding may be "active" or "enhanced"[11]; we did not specify which of these we include under our general category of increased case-finding, as this will vary according to local conditions. In this study, the utility of using PIMs to describe the population impact of the directly observed component of DOTS was compared with that of increased case-finding, using India, where TB provides a major health burden, as an example[13].

## Methods

To describe the impact of preventive and treatment interventions, the number of events prevented in a population (NEPP) is defined as "the number of events prevented by the intervention in your population over a defined time period" [2]. NEPP extends the number needed to treat (NNT) beyond the individual patient to a specific population. The components of the calculations (see Appendix for the formulae) are: (i) population denominator (size of the population); (ii) proportion of the population with the disease; (iii) the incremental proportion of the diseased population eligible for the proposed intervention (the latter requires the actual or estimated proportion who are currently receiving, and are compliant with, the interventions, subtracted from best practice goal from guidelines or targets); (iv) baseline risk (the probability of the outcome of interest in this or similar populations); and the(v) relative risk reduction (RRR) associated with the intervention. Confidence intervals (CIs) for the measures were obtained using simulation methods . The calculations were made using published data (each source is referenced in the Results section).

Data from the paper by Baltussen et al[14] were used to calculate the costs, which are reported in international dollars (I$). Direct observation of therapy requires 40 clinic visits for supervision (24 in the intensive and 16 in the continuation phase) rather than 4 (1 initial and 3 for progress monitoring) for unobserved treatment. Each clinic visit costs I$3.85. Each TB smear test for those identified by case finding costs I$1.14, and we multiplied this by the prevalence of symptoms in the population (to reflect that increased case-finding would occur among those who are symptomatic). We do not have an estimate of the costs of detecting those who are symptomatic in order for them to have a sputum smear for diagnosis, as the way in which cases are detected will differ depending on the setting and on whether the case finding is "active" or "enhanced"[11]. However, we doubled the cost of the smears to allow for the costs of the case finding to detect symptomatic people who will subsequently have a smear, and also added the treatment costs for the cases of smear-positive TB identified by the case-finding process (drug costs at I$7.84 plus four clinic visits per patient).

## Results

The published data show that 45% of identified TB patients in India are covered by the DOTS programme[15] However, only an estimated 50% of smear-positive TB cases in India are identified by the current passive case-finding approach[13]. The benefit of increasing this by a further 20% (or 40% of the currently unidentified cases) was estimated, as this is consistent with the national and global target of detecting 70% of smear-positive cases[6,13], and is a conservative estimate of the proportion of undetected cases that might be found through case finding[11,16]. The added benefit of the directly observed component of DOTS on cure and completion rates has been estimated in a systematic review as 6%, which is the figure used in our study, although this was not statistically significantly different from no benefit[12]. We assumed that this benefit would translate to a similar reduction in case fatality. Six-month case fatality for treated TB was estimated in the original MRC trial of the treatment of TB as 7.3% and for untreated TB 27%, with a reduction of 73% from treatment,[17] and are consistent with a reported global case fatality rate of 23% [9,18]. We also assumed that only 71% of patients with TB would complete treatment in the absence of the DOTS programme, taken from the "cure plus treatment completion" figures of the control groups in the Cochrane review[12]. This is consistent with the 76% quoted elsewhere [19] Table 1 demonstrates that the current direct observation of the DOTS programme would, on average, produce a reduction of 0.188 deaths from TB for each 100 000 people in the population in a year. If the identification rate of TB was increased by 20%, using increased case-finding, and 71% of these people completed treatment, there would be a reduction of 1.79 deaths from TB for each 100 000 people in the population in a year, nearly 10 times the numbers of deaths saved by direct observation of therapy.

### Costs

The cost of direct observation for 36 extra clinic visits is I$138.6 per patient. Table 1 shows that there are 43 smear-positive cases per 100 000 population in a year, thus the cost of direct observation for these 43 cases is I$5960, resulting in a cost of I$31702 per life saved.

For case finding, we estimated that 2% of the population may be symptomatic (from previous figures of 1.2–6.7% symptomatic people in possibly similar populations[11,16]), hence requiring 2000 smears. This may be a conservative estimate, as Baltussen estimates that only 30 smears need to be taken to find one case, whereas we estimated it would take 2000 smears to find 12 cases (40% of the 32 undiagnosed in our population of 100 000), or 167 smears to find one case. The drug costs and 4 clinic visits total I$23.24 for each of the 12 identified cases. The cost of case finding is thus estimated to be I$4839, or I$2703 per life saved (table 2).

## Discussion

This study shows that, given the parameters used, increasing case finding by 20% would lead to nearly 10 times the numbers of deaths saved by the current direct observation of therapy in India. This is due to a greater RRR, applied to a higher baseline case-fatality, hence producing a larger absolute benefit. The analysis clearly shows that, based on the assumptions made, improving case-finding for TB will save far more lives than maximizing the use of the directly observed component of DOTS among currently identified cases in India. This has obvious implications for health decision-makers. Although the DOTS program does encourage better case detection, it does not include active or enhanced case-finding [5,6,20]. Our findings concur with those who feel that case finding should receive more attention [7,21,22], and that a careful approach to examining the benefits of different aspects of DOTS programmes should be encouraged[5].

There are several different strategies for increasing case-finding, and the choice should depend on local factors such as disease prevalence, adequacy of training of healthcare workers and willingness of the infected population to seek care for symptoms[11]. The benefits of active, passive or enhanced case-finding among general, high-risk, and symptomatic populations have been reviewed in depth by Golub et al[11].

The calculations made in this study depend on the accuracy of the data used. Each estimate is subject to potential error, and ideally, each population should obtain its own data in order to produce accurate estimates of population impact of risks and interventions. The literature-based estimates of RRR are also open to question. Our use of the baseline risk of death from treated and untreated TB uses old trial data, although the treated risk is consistent with recently published data of death rates of 4.4% among DOTS-registered cases in countries with high TB burden[13]. We applied the cure rate of the systematic review of DOTS therapy to the case fatality, and this may be open to question. An updated Cochrane Review was published in 2006[23], which came to the same conclusion about a non-significant 6% difference in the outcome cured or completed treatment. There were insufficient numbers to allow analysis of case fatality, although four of the included trials did include mortality as an outcome measure. The results of the calculations depend on the various assumptions made. For example, changing the estimate of TB treatment completion would have changed the number of deaths prevented, but is unlikely to have changed the ranking of benefit between increasing case-finding and direct observation of treatment. Thus it is important to obtain relevant local data, apply the approach to different populations and population subgroups, and to test the robustness of the estimates to varying the assumptions. For example, in a population where case finding is already extensive, costs per case detected are likely to be higher, and thus maximizing the use of directly observed therapy could have the greater impact.

The confidence intervals were made taking into account the potential variability of the estimates used; however, changing the estimates used for each of the components of the PIMs themselves has additional potential to influence the results. This can be explored by recalculating the measures using different estimates as appropriate to particular local settings. To assist potential users, we have developed a public access website that automatically calculates both the PIMs and their confidence intervals .

We did not include the impact of transmission dynamics on the rest of the population in our calculations, as this would add considerably to the complexity of the approach. In terms of the comparison between direct observation and improved case-finding, the lack of attention to transmission dynamics will underestimate the relative benefit of improved case-finding, as the resulting early intervention is likely to be more effective than the later attempt to improve therapy adherence. The decision to restrict the analysis to smear-positive cases was based on the better accuracy of this measure of disease burden, although it will underestimate the benefit of treatment on extrapulmonary TB. We do not wish to claim false accuracy for the results presented; however, they do use the best available data, are easy to compute and produce results that are easy to understand. The level of accuracy should be adequate for most policy decisions, and is preferable to making decisions in the absence of estimates of population benefit.

If PIMs prove useful where relevant data exist, then this could stimulate better collection and use of health data in situations where there are currently too few data to enable reasonable calculations. Similarly, the use of PIMs with confidence intervals enables policy-makers to be explicit about the uncertainties they face.

The measures can be used to compare between populations, in which case standardization for age, sex, ethnicity and socioeconomic status may be required. The measures can also be used to compare between segments of a population, in which case the potential for an intervention to reduce health inequalities and increase equity within a population can be explored[3].

For ease of presentation, the population denominator we used was 100 000 adults; however, one of the main benefits of these measures is the ability to relate to local context, hence policy-makers can make the calculations for their own population denominator.

The addition of information on costs to the population impact measures will also be important for policy makers, and our estimated costs for the programme will allow costs and their consequences to be calculated[24]. PIMs differ from measures used in cost-effectiveness analyses by not including life expectancy or the utility or valuation of the health outcome. They thus produce outcomes expressed as events rather than generic outcomes such as QALY or DALY gained[25]. We have previously suggested that the valuation of the events prevented should be performed by the policy-maker in relation to the costs of the intervention, once the measures have been produced [26]. Although our cost estimates are prone to error, they were derived using previously published costs applied to this simple model of health gain. They show that the programme costs for the direct observation of therapy and the detection of new cases are similar, although the health gain is much greater for the detection of new cases. In fact, our estimate of the health gain from direct observation may be an overestimate, as the relative risk quoted was not significantly different from no effect.

The data shown on TB control demonstrates the potential benefit to policy-makers from the use of PIMs. There is some evidence that the way in which health benefits are framed impacts on the policy decisions reached[27], although there is debate about the consistency of such an effect[28]. We previously showed that although clinicians were more influenced by benefit expressed as RRR than as PIMs[29], public health professionals were more likely to prioritise interventions based on the use of PIMs than on the basis of more complex demonstrations of health gain [26]. The evidence base on what are the most effective methods of presenting health gains from interventions in order to assist in policy-making is, however, weak. Health policy requires more than merely demonstration of health gain [30-32], as the complexity of policy-making includes social and political drivers of decision-making, as well as the need to take into account issues such equity, total budget impact, total morbidity and disease severity.

Our method is considerably simpler than other modelling exercises that have also examined the potential benefits of case finding[7,8], and may compensate for this simplicity by the increased ease of when making the calculations and understanding the results. Both modelling and the calculation of PIMs have similar reliance on data availability and accuracy. Our method is not intended to replace more complex analyses of TB control[14], nor is it intended to reflect adversely on the DOTS programme, which has many components, of which direct observation is only one. It is intended to show how to use local data to provide simple demonstrations of health benefit, which can then be used in policy-making. We believe that it is worth testing the hypothesis that simple demonstration of the population health-gain consequences of interventions will lead to the introduction of health policies that can provide appropriate priorities to improve health in developing countries. This could be of marked benefit in TB, a major threat to global health.

## Conclusion

Increasing case-finding for TB will save nearly 10 times more lives than will the use of the directly observed component of DOTS in India, at a smaller cost per life saved. The demonstration of the population impact, using simple and explicit numbers, may be of value to policy-makers as they prioritise interventions for their populations.

## Appendix

Formula for calculating population impact measure

### Number of events prevented in your population (NEPP) [2]

*NEPP *= n* *P*_d _* *P*_e _* *r*_u _* *RRR*

where *n *= population size, *P*_*d *_= prevalence of the disease in the population, *P*_*e *_= proportion eligible for treatment, *r*_*u *_= risk of the event of interest in the untreated group or baseline risk, and *RRR *= relative risk reduction associated with the treatment

In order to reflect the incremental effect of changing from current to "best" practice and to adjust for levels of compliance the proportion eligible for treatment *P*_*e *_is

((*P*_*b *_- *P*_*t*_) * *P*_*c*_

where *P*_*t *_is the proportion currently treated, *P*_*b *_is the proportion that would be treated if best practice was adopted and *P*_*c *_is the proportion of the population that is compliant with the medication.

## Competing interests

The author(s) declare that they have no competing interests.

## Authors' contributions

RFH, RE and IB were involved in the design and conception of the study, IG and IB in statistical analysis, RFH and SA in data acquisition, RFH, IG, IB and JAV in analysis and interpretation, RFH and IG in initial drafting and all authors in critical revision of the manuscript.

## Funding

The authors received no funding for this study.

## Pre-publication history

The pre-publication history for this paper can be accessed here:



## Figures and Tables

**Table 1:** Number of deaths prevented among 100,000 people in India with direct observation of therapy and increased use of case finding

	Direct observation of therapy	Increase in TB case-finding
Population denominator	100 000	100 000
Incidence of smear-positive TB	0.00043*	0.00032†
Proportion of the diseased population eligible for the intervention: best practice goal minus current practice levels	All those identified are estimated to be subject to direct observation	0.40 (0.284)‡
Baseline risk (case fatality)	7.3% [17]	27% [17]
RRR from intervention	0.06 [12]	0.73 [17]
NEPP (deaths from TB prevented in the next year)	0.188§ (95% CI 0.014–0.294)	1.79¶ (95% CI 1.01–2.69)

NEPP, number of events prevented in the population; RRR, relative risk reduction; TB, tuberculosis.

The examples are based on a population of 100 000, but in reality the actual number and composition of the population to which the results are to be applied would be identified.

*Based on 57% DOTS case detection rate, or 43 per 100 000 per year [13]

†The estimated true incidence rate is 75 per 100 000[13], so 32 per 100 000 is the potential for case finding.

‡40% of cases identified by case detection; proportion of identified cases who will complete treatment = 71%, thus 0.40 × 0.71 = 0.284.

§100 000 × 0.00043 × 0.073 × 0.06 = 0.188.

¶100 000 × 0.00032 × 0.284 × 0.27 × 0.73 = 1.79.

**Table 2:** Costs of direct observation of therapy and increased use of case finding among 100 000 people in India

	Direct observation of therapy	Increase in TB case-finding
Extra TB smears for case detection (@I$2.28)	N/A	2000
Number of extra clinic visits (@I$3.85)	36 per patient	4 visits for each of 12 new patients identified
Extra drug costs (@I$7.84)	N/A	For each of 12 new patients identified
Total cost (I$)	5960	4839
Cost per life saved (I$)	31702	2703

I$, international dollars; N/A, not applicable; TB, tuberculosis.
